# Postoperative Changes in Aqueous Monocyte Chemotactic Protein-1 Levels and Bleb Morphology after Trabeculectomy vs. Ex-PRESS Shunt Surgery

**DOI:** 10.1371/journal.pone.0139751

**Published:** 2015-10-01

**Authors:** Kohei Shobayashi, Toshihiro Inoue, Motofumi Kawai, Keiichiro Iwao, Saori Ohira, Sachi Kojima, Utako Kuroda, Kei-Ichi Nakashima, Hidenobu Tanihara

**Affiliations:** 1 Department of Ophthalmology, Faculty of Life Sciences, Kumamoto University, Kumamoto, Japan; 2 Department of Ophthalmology, Asahikawa Medical University, Asahikawa, Japan; Bascom Palmer Eye Institute, University of Miami School of Medicine;, UNITED STATES

## Abstract

**Purpose:**

To evaluate the postoperative changes in blebs and levels of aqueous monocyte chemotactic protein-1 (MCP-1) after trabeculectomy vs. Ex-PRESS tube shunt surgery.

**Methods:**

Rabbits were subjected to trabeculectomy or Ex-PRESS tube shunt surgery and observed for up to 3 months. Intraocular pressure (IOP) was measured using a rebound tonometer. The MCP-1 level was measured by enzyme-linked immunosorbent assay (ELISA). Bleb morphology was evaluated using photos and anterior-segment optical coherence tomography (OCT).

**Results:**

There were no differences in bleb appearance or IOP at any time between the groups. Bleb wall density in the anterior-segment OCT image was significantly lower 1 week after surgery in the Ex-PRESS group than the trabeculectomy group. The MCP-1 level in control eyes was 304.1 ± 45.2 pg/mL. In the trabeculectomy group, the mean aqueous MCP-1 level was 1444.9, 1914.3, 1899.8, 516.4, 398.3, 427.3, 609.5, 1612.7, 386.2, and 167.9 pg/mL at 3, 6, and 12 h, and 1, 2, 5, 7, 14, 30, and 90 days after surgery, respectively. In the Ex-PRESS group, the corresponding values were 1744.0, 1372.0, 932.5, 711.7, 396.1, 487.3, 799.5, 1327.9, 293.6, and 184.0 pg/mL. There were no significant differences in the aqueous MCP-1 level between the groups at any time point.

**Conclusion:**

The postoperative changes were similar in the Ex-PRESS and trabeculectomy groups, except for bleb wall density in the anterior-segment OCT image. The postoperative aqueous MCP-1 level had bimodal peaks in both groups.

## Introduction

Trabeculectomy is the surgical method used most commonly to lower intraocular pressure (IOP) in glaucoma [1–2]. Recently, the use of glaucoma drainage devices (GDD) has increased [1–2], and their IOP-lowering effects are comparable to that of trabeculectomy [3–4]. Although the GDD might alter the pathophysiology differently from trabeculectomy, and is dependent on the implant device, the differences are not fully understood. Of the various GDDs, the Ex-PRESS device is a unique valveless stainless steel tube, which is implanted under a scleral flap, causing bleb formation around the flap. Consequently, the wound-healing process and surgical results of Ex-PRESS shunt surgery have been similar to those of trabeculectomy in randomized controlled trials [5–9].

Monocyte chemotactic protein-1 (MCP-1; also known as CCL1) is a potent chemotactic factor for monocytes that is involved in inflammatory cascades and fibrotic changes [10]. MCP-1 is thought to be involved in the tissue infiltration of monocytes and T cells in a variety of inflammatory diseases, suggesting that elevated levels of MCP-1 in the aqueous humor contribute to the scarring of filtration blebs. Previously, we reported that small-incision phacoemulsification was a risk factor for a poor surgical outcome in a mitomycin-C trabeculectomy in eyes with open-angle glaucoma [11–12], and the aqueous MCP-1 level was elevated in those patients and a rabbit model [13–14]. Furthermore, the aqueous MCP-1 level in phakic eyes with open-angle glaucoma predicted the results of trabeculectomy [15]. Therefore, aqueous MCP-1 might be involved in the inflammatory response and scarring process after filtration surgery for glaucoma. As far as we know, however, no study has investigated the changes in the level of aqueous humor MCP-1 after GDD. Since Ex-PRESS shunt surgery can omit iridectomy, we hypothesized that the aqueous MCP-1 could be lower compared to trabeculectomy at some time point after surgery.

## Methods

### Animal Experiment

Experiments were conducted according to the guidelines of the ARVO Statement for the Use of Animals in Ophthalmic and Vision Research and approved by the Animal Use Committee of Kumamoto University. The study used 126 female Japanese white rabbits (2.0–3.0 kg, 12–14 weeks old). Sixty animals underwent trabeculectomy, another sixty underwent Ex-PRESS shunt surgery, and six animals were examined without surgery as controls. The animals subjected to surgery were euthanized after collecting aqueous humor 3, 6, and 12 h and 1, 2, 5, 7, 14, 30, and 90 days after surgery (n = 6 each) and the six control animals were euthanized without surgery. IOP was measured preoperatively and before collecting aqueous humor using TONOVET (Icare, Helsinki, Finland). The measurement was made before intramuscular injection of anesthesia.

#### Trabeculectomy Protocol

The animals were anesthetized with an intramuscular injection of ketamine hydrochloride (Ketalar; 25 mg/kg body weight; Daiichi Sankyo, Tokyo, Japan) and xylazine hydrochloride (Celactal; 10 mg/kg body weight; Bayer Medical, Leverkusen, Germany). The right eye was washed with topical povidone iodine and a lid speculum was placed to hold the lids open. Then, a 7 mm conjunctival incision was made along the superior corneal limbus with spring micro-scissors to make a fornix-based conjunctival flap. A partial-thickness, trapezoidal-base, 3 mm scleral flap was created in the superior sclera, hinged at the limbus under the conjunctival flap. The scleral flap was dissected forward until it reached under the limbus. A sponge soaked with mitomycin C (MMC; 0.4 mg/mL; Kyowa Hakko Kirin, Tokyo, Japan) was applied to the scleral flap and subconjunctival tissues for 3 min. Then, the MMC was washed out with normal saline (10 mL; Otsuka, Tokyo, Japan). An ophthalmic straight knife (MANI, Utsunomiya, Japan) was inserted into the anterior chamber from underneath the scleral flap, creating an ostomy. The entrance to the anterior chamber was enlarged using a Kelly Descemet membrane punch (Inami, Tokyo, Japan). Forceps were used to pull the peripheral iris through the ostomy site and scissors were used to create the peripheral iridectomy. Using 10–0 nylon (MANI), two sutures were placed in the corner of the trapezoid that was not a hinge to close the scleral flap. Then the conjunctival peritomy was closed using 10–0 nylon sutures. At the end of the surgery, ofloxacin ointment (Tarivid; Santen, Osaka, Japan) was applied. No additional medications were given postoperatively.

#### Ex-PRESS Shunt Surgery Protocol

The Ex-PRESS shunt devices were provided from Alcon Japan (Tokyo, Japan). The procedure was the same as the trabeculectomy described above until the MMC was washed out. Afterward, a 25-gauge needle (25G MVR knife; MANI) was inserted into the anterior chamber from underneath the scleral flap. Then the Ex-PRESS shunt device (Alcon Japan) was placed in the anterior chamber through the ostium created by the needle, following the manufacturer’s protocol. After fixation of the Ex-PRESS device, the scleral flap and conjunctival incision were sutured with 10–0 Nylon, as in the trabeculectomy described above. Ofloxacin ointment was also applied. No additional medications were given postoperatively.

#### Bleb Photo and Anterior-segment OCT Imaging

A photo was taken of each bleb with a digital camera (CX4; RICOH, Tokyo, Japan), and its area and vascularity at 1, 2, 4, and 12 weeks postoperatively were classified using the Moorfields Bleb Grading System (http://blebs.net/) by three independent researchers who were blind to the condition of the bleb (n = 3, each).

Anterior-segment OCT images were acquired using CASIA^®^ (Tomey, Nagoya, Japan) 2, 5, 7, and 14 days postoperatively (n = 3, each), and the intensity of the bleb wall in the horizontal image was measured using CASIA bleb assessment software ver. 4.0L (Tomey).

#### Measuring the Aqueous MCP-1 Level

Aqueous humor was collected from the rabbit eyes under anesthesia using the method described above. In total, 100–150 μL aqueous humor was gently withdrawn. Contact with the intraocular tissues was carefully avoided and contamination of the samples with blood was prevented. After centrifugation at 7830 g for 5 min at 4°C, a 50 μL sample was immediately stored at –80°C.

The concentrations of MCP-1 in the aqueous humor of the rabbits were measured with an enzyme-linked immunosorbent assay (ELISA; WLS−E90087Rb; USCN Life Science, Wuhan, China). The absorbance was read at 450 nm in a microplate reader (Multiskan FC; Thermo Fisher Scientific, Yokohama, Japan). The detection range was 15.6–1,000 pg/mL. To ensure that there was sufficient material for the experiment, the aqueous humor was diluted four times with phosphate-buffered saline (D-PBS[–]; Wako, Osaka, Japan).

#### Immunohistochemistry

Immunohistochemistry analysis has been described previously [13]. Briefly, the enucleated eyes were fixed in Super Fix (KY-500; Kurabo, Osaka, Japan) for 48 h at 4°C, treated in 30% sucrose (Wako) overnight at 4°C, embedded in OCT compound (#4583; Sakura Finetek, Torrance, CA, USA), and cut into vertical frozen sections (8 μm thick). For eyes subjected to Ex-PRESS tube shunt surgery, the sections were made at a site close to the implant, because our instrument could not cut the implant. After blocking peroxidase with 3% H_2_O_2_ in methanol, the sections were treated with 10% normal goat serum for 30 min at room temperature (RT), and incubated with goat anti-α-SMA antibody (1:100; Dako, Kyoto, Japan) for 30 min at RT. For dual labeling, the sections were washed and incubated with affinity-purified, biotin-conjugated donkey anti-goat IgG secondary antibody (1:500; EMD Millipore, Temecula, CA, USA) for 2 h at RT and then washed and incubated with detection reagents (Simple Stain Rat MAX-PO [M]; Nichirei) for 30 min at RT. As a chromatogen, 3,3’-diamino-benzidine tetrahydrochloride (DAB Peroxidase Substrate Kit SK-4100; Vector) was used. Hematoxylin was used as a counterstain. As a negative control, adjacent sections were incubated without primary antibodies. The slides were observed using a fluorescence microscope (BZ-X710; Keyence Japan, Osaka, Japan), and some of the acquired photos were combined using software (BZ-H3A; Keyence).

### Statistical Analysis

IOP and concentrations of MCP-1 were compared between the trabeculectomy and Ex-PRESS groups using the Wilcoxon rank-sum test. Postoperative IOP and concentrations of MCP-1 were analyzed using Dunnett’s test, using the preoperative/no-surgery values as respective controls. P-values less than 0.05 were considered significant.

## Results

### Aqueous MCP-1 Level after Trabeculectomy vs. Ex-PRESS Shunt Surgery

**Fig 1:**
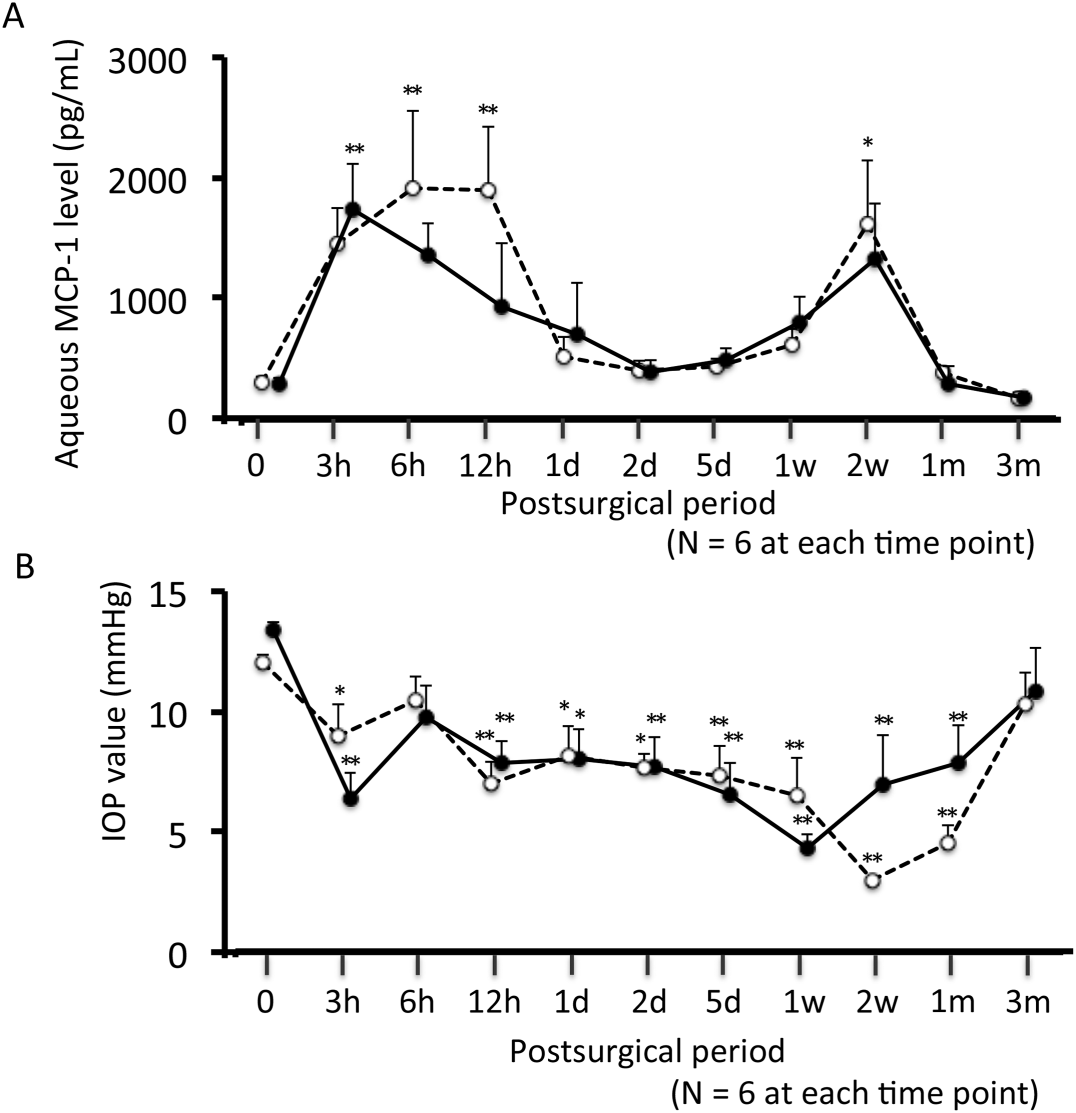
Time course of aqueous MCP-1 (A) and IOP level (B). Open circles with dotted line, trabeculectomy group; closed circles with solid line, Ex-PRESS tube shunt surgery group. Mean ± standard error. *P < 0.05, **P < 0.01, Dunnett’s test compared to non-operated eyes.

Fig 1A shows the time course of the aqueous MCP-1 level after surgery; there were bimodal peaks in both groups. The mean (±SE) MCP-1 level was 304.1 ± 45.2 pg/mL in controls. It was 1444.9 ± 300.0, 1914.3 ± 645.2, 1899.8 ± 515.0, 516.4 ± 158.2, 398.3 ± 73.1, 427.3 ± 65.7, 609.5 ± 124.2, 1612.7 ± 537.2, 386.2 ± 31.0, and 167.9 ± 45.0 pg/mL at 3, 6, 12, 24, and 48 h, and 5, 7, 14, 30, and 90 days after surgery, respectively, in the trabeculectomy group, and 1744.0 ± 376.6, 1372.0 ± 253.1, 932.5 ± 531.7, 711.7 ± 427.2, 396.1 ± 92.7, 487.3 ± 101.3, 799.5 ± 227.0, 1327.9 ± 466.0, 293.6 ± 148.6, and 184.0 ± 25.4 pg/mL in the Ex-PRESS group. There were no significant differences at any time point between the two groups (P = 0.631, 1.000, 0.109, 0.631, 0.749, 0.749, 0.631, 0.873, 0.078, and 0.423, respectively). At 6, 12 h and 14 days after surgery, the MCP-1 level was significantly higher in eyes subjected to trabeculectomy than in controls (P = 0.006, 0.007, and 0.039, respectively). Conversely, the level was significantly higher in eyes that underwent shunt surgery than in control eyes only at 3 h after surgery (P = 0.010).

### Pre- and Postoperative IOP

Fig 1B shows the time course of IOP after surgery. The preoperative mean (±SE) IOP in the trabeculectomy and shunt surgery groups was 12.05 ± 0.29 and 11.93 ± 0.31 mmHg, respectively (n = 60 per group). The postoperative IOP was measured before collecting aqueous humor; 3, 6 and 12 h after surgery; and 1, 2, 5, 7, 14, 30, and 90 days after surgery (n = 6 per time point). In the trabeculectomy group, the postoperative mean (±SE) IOP at the respective times was 9.00 ± 1.29, 10.50 ± 0.96, 7.00 ± 0.93, 8.17 ± 1.22, 7.67 ± 0.61, 7.33 ± 1.23, 6.50 ± 1.57, 3.00 ± 0.00, 4.50 ± 0.81, and 10.33 ± 1.28 mmHg. In the shunt surgery group, the respective postoperative pressures were 5.67 ± 0.95, 8.67 ± 1.23, 7.00 ± 0.82, 7.17 ± 1.08, 6.83 ± 1.11, 5.83 ± 1.19, 3.83 ± 0.54, 6.17 ± 1.83, 7.00 ± 1.37, and 9.67 ± 1.63 mmHg. There were no significant differences in the IOP between the two groups at any time point. In both groups, the IOP decreased significantly after surgery, except at 6 h and 90 days.

### Bleb Appearance and Anterior-segment OCT Images

**Fig 2:**
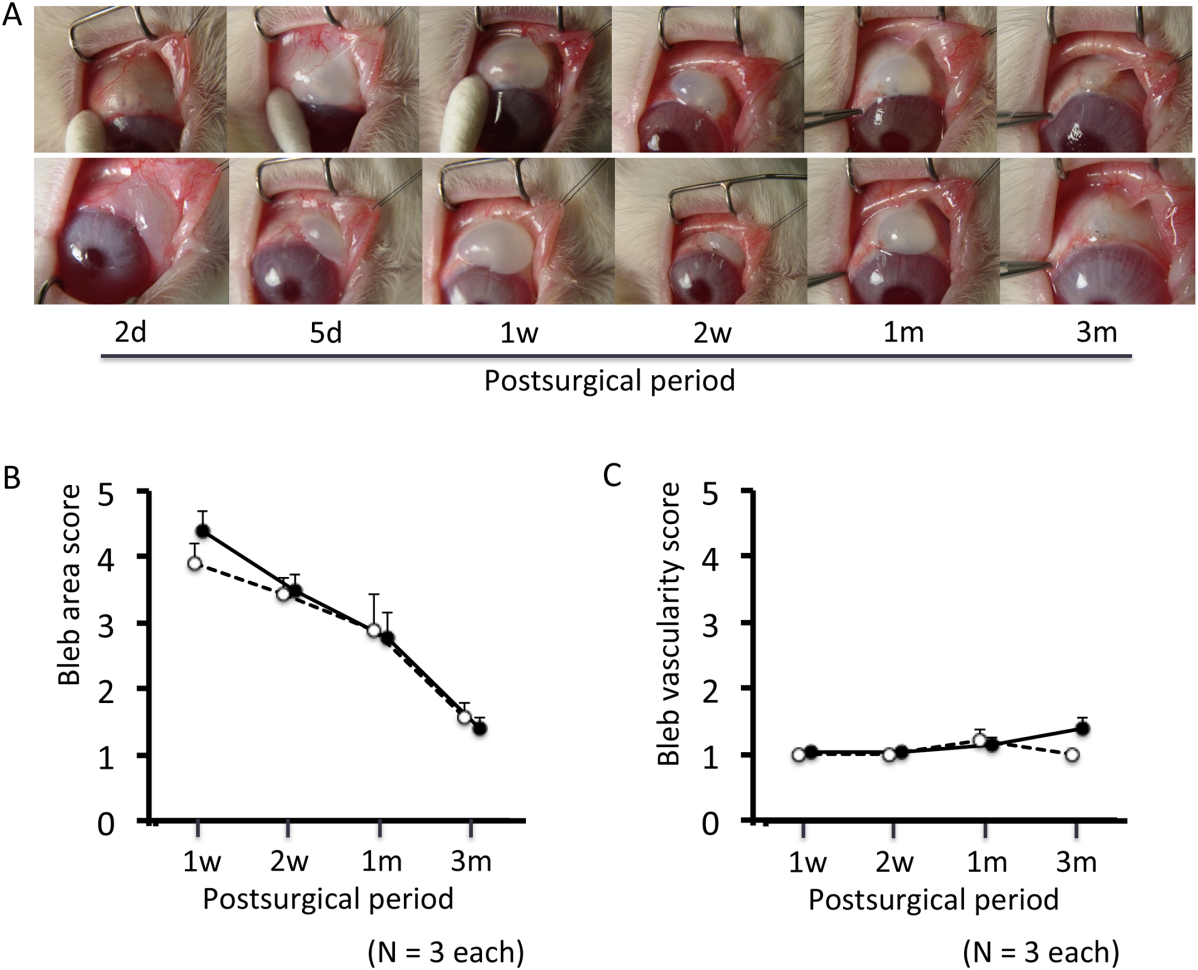
Time course of bleb appearance. (A) Representative photos after trabeculectomy (top) and Ex-PRESS tube shunt surgery (bottom). (B, C) Blebs were scored using the Moorfields Bleb Grading System. Open circles with dotted line, trabeculectomy group; closed circles with solid line, Ex-PRESS tube shunt surgery group. Mean ± standard error.

**Fig 3:**
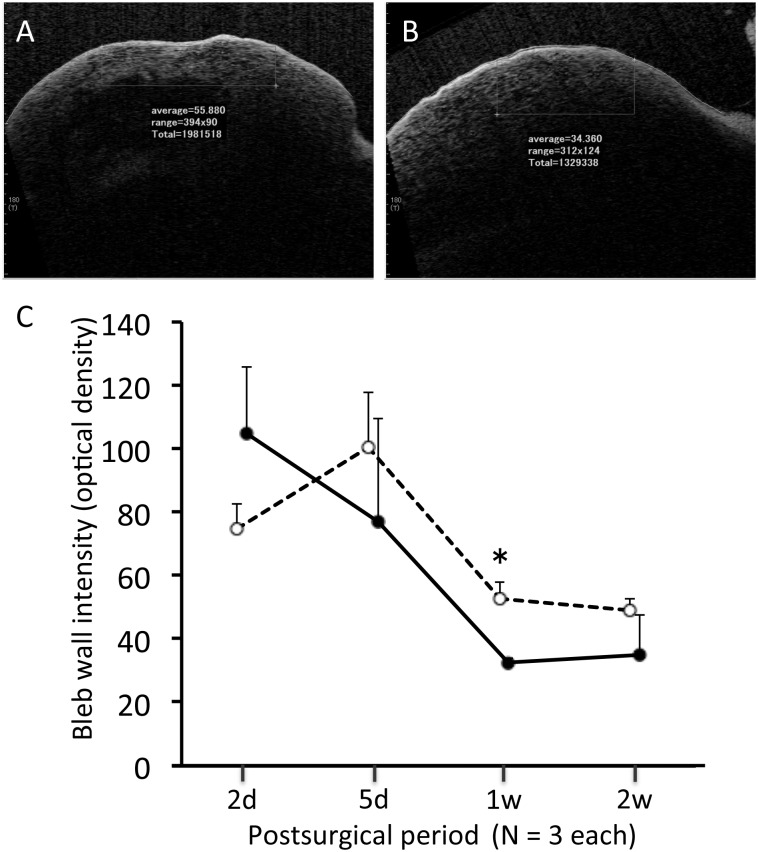
Intensity of the bleb wall measured using anterior-segment optical coherence tomography. Representative images in a horizontal scan 1 week after trabeculectomy (A) and Ex-PRESS tube shunt surgery (B). Time course of intensity. Open circles with dotted line, trabeculectomy group; closed circles with solid line, Ex-PRESS tube shunt surgery group. Mean ± standard error. *P < 0.05 by Wilcoxon rank-sum test.

Representative blebs are shown in Fig 2A. Similar avascular cystic blebs were observed in the groups. In both groups, the bleb became flat by 3 months after surgery. There were no significant differences in area or vascularity between the groups at any time point after surgery (Fig 2B and 2C). In all eyes after shunt surgery, the device remained attached to the iris at all time points. In the AS-OCT images (Fig 3), only 3 (12.5%) of 24 eyes had a fluid-filled space, and many of the blebs were filled with sponge-like tissue, followed by a vague shadow of the scleral bed. This made it difficult to compare the imaging characteristics (i.e., height and wall thickness of the bleb, height of the fluid-filled cavity, filtration opening in the scleral flap) between the groups. However, the density of the bleb wall was significantly lower 1 week after surgery in the Ex-PRESS group than the trabeculectomy group (P < 0.05). Two days after surgery it was significantly denser than 7 and 14 days postoperatively in the Ex-PRESS group and at 14 days in the trabeculectomy group, suggesting a more rapid decrease in the Ex-PRESS group than in the trabeculectomy group.

### Immunohistochemical Analysis of α-SMA

**Fig 4:**
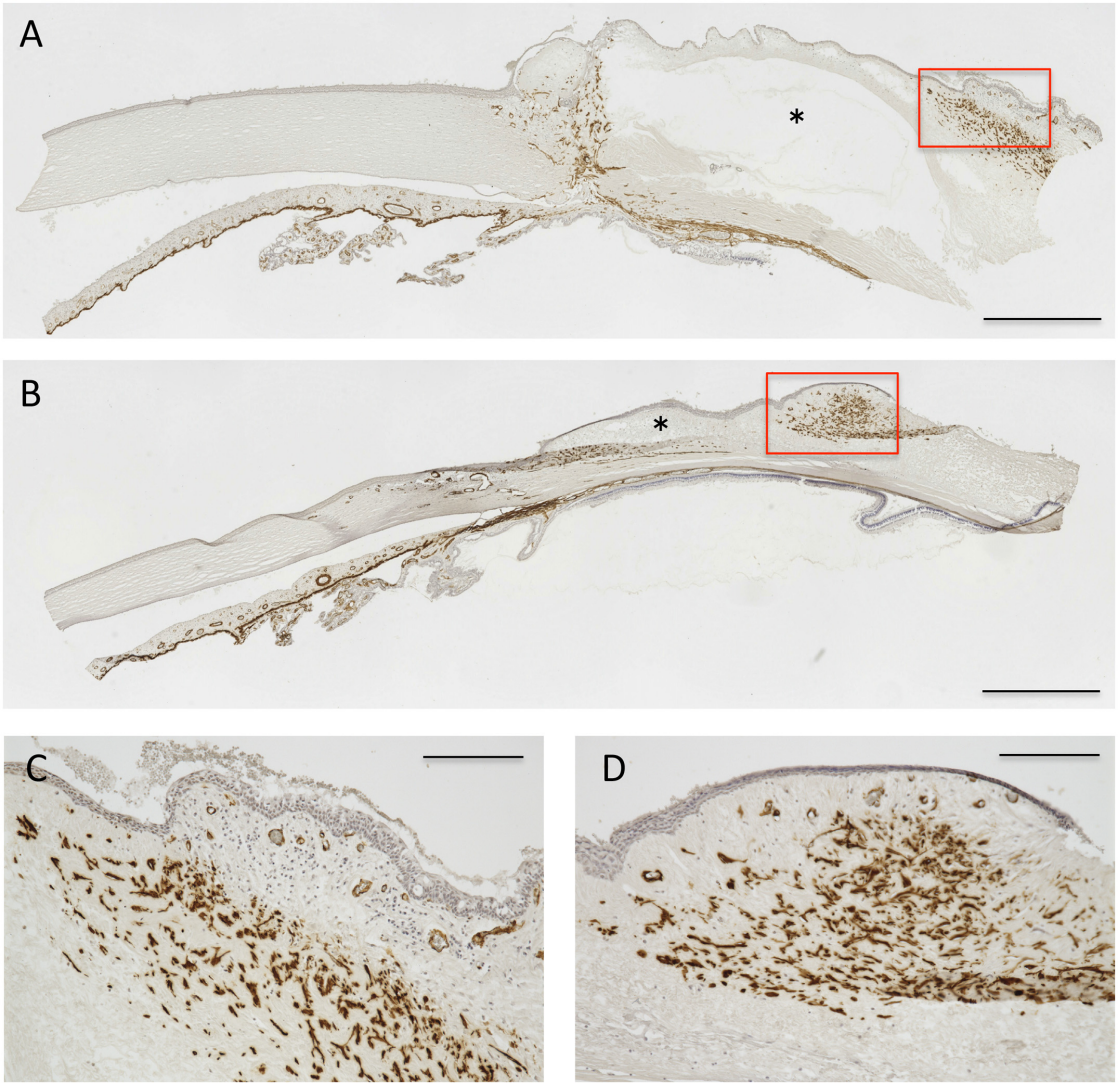
Immunohistochemical analysis. α-SMA-positive cells were detected as brown cells in the bleb after trabeculectomy (A, C) and Ex-PRESS tube shunt surgery (B, D). The red squares in A and B outline C and D (higher magnification), respectively. Scale bar, 1 mm (A, B) and 200 μm (C, D).

Generally, the amount of α-SMA positive cells represents the extent of tissue scarring at the time point. The immunohistochemical analysis showed immunoreactivity for α-SMA in cells around the fluid cavity in the bleb, suggesting that the conjunctiva cells partially transdifferentiated into myofibroblast-like cells (Fig 4). Qualitatively, there were no significant differences in the distribution of α-SMA-positive cells between the trabeculectomy and Ex-PRESS groups.

## Discussion

Ex-PRESS tube shunt surgery has several advantages over conventional trabeculectomy. First, the device does not require a sclerostomy or peripheral iridectomy; therefore, it induces a smaller postoperative inflammatory reaction and causes less hyphema [7,9,16–18]. This might also lead to more rapid recovery of vision after Ex-PRESS tube shunt surgery compared to trabeculectomy [9,17,19,20]. Second, the operative procedure for the shunt surgery is relatively easy to standardize, and it provides more stable aqueous outflow from the anterior chamber to the space under the scleral flap. Subsequently, the variance in IOP after such surgery is lower [9], and the ratio of postoperative hypotony is also lower compared to trabeculectomy [7,16–18]. Despite these advantages, the control of postoperative IOP and success rates are similar between the shunt surgery and trabeculectomy [9,16–19]. Therefore, some subclinical mechanisms might offset the advantages described above, leading to the similar surgical success rates.

In the present study, the blebs in both study groups had the same appearance and tended to become flat within 3 months of surgery. One retrospective study reported that bleb height was lower in an Ex-PRESS group in the first 3 months postoperatively, and the blebs were more diffuse after 3 to 18 months compared to a trabeculectomy group [17]. Because they also used the Moorfields Bleb Grading System, but conducted the study on human, the conflict in bleb appearance between our study and that study might result from the different species studied.

To the best of our knowledge, no study has compared anterior-segment OCT images between Ex-PRESS tube shunt surgery and trabeculectomy. OCT provides more quantitative data on the internal structures of blebs than a slit-lamp examination, and is useful for understanding the postsurgical mechanisms of bleb failure. Our study compared anterior-segment OCT bleb images in a limited number of eyes over a limited period. Although our data are not conclusive, they suggest that there is a difference in bleb wall density between trabeculectomy and Ex-PRESS tube shunt surgery. Because the internal structures of blebs observed with anterior-segment OCT reflect future control of IOP [21–22], further investigation of this is required in clinical cases.

In the present study, the IOP was lower until 1 month after surgery, and then it returned to the preoperative level in both groups. The time-dependent changes in the two groups were similar. In previous clinical studies, the postoperative IOP control and success rates were similar between Ex-PRESS tube shunt surgery and trabeculectomy [9,16–19]. Supporting this, in the present study, there were no significant differences in immunohistochemical analysis of the filtering bleb between the groups. Therefore, despite differences in the species studied, our results using rabbits are comparable to clinical studies.

The chemotactic factor MCP-1 is involved in tissue infiltration of monocytes and T cells in a variety of inflammatory diseases. Previously, we reported that the aqueous MCP-1 level in phakic eyes with open-angle glaucoma predicted the results of trabeculectomy.^15^ Therefore, the aqueous MCP-1 might be involved in the inflammatory response and scarring process after filtration surgery for glaucoma. Postoperatively, the time-course of the aqueous MCP-1 level showed bimodal peaks (at 3–12 h and 2 weeks) in both groups, which was unexpected. Previously, we measured the MCP-1 concentration after phacoemulsification in the aqueous humor of rabbit eyes, and found that the level 30 days after surgery was significantly higher than the preoperative level (207.1 ± 62.9 vs. 31.2 ± 12.5 pg/mL). In this study, a preliminary experiment revealed marked MCP-1 elevation 12 h after phacoemulsification, but the level returned to near basal levels within several days (data not shown), which represented a unimodal peak in the acute phase. As the first peak in the aqueous MCP-1 level was observed at a similar postoperative time in both eyes after filtration surgery and phacoemulsification, while the second peak 2 weeks after surgery occurred only in eyes after filtration surgery, different postoperative inflammatory reactions may have occurred in the anterior chamber in eyes after filtration surgery compared to after phacoemulsification.

The mechanism underlying the bimodal peak in the aqueous MCP-1 after filtration surgery is unclear. The general wound-healing process is divided into four phases: hemostasis, inflammation, proliferation, and remodeling [23]. The first peak was at 3 h in the Ex-PRESS group and at 6–12 h in the trabeculectomy group, and this might be explained by the migration of inflammatory cells in the inflammatory phase of wound healing. The second peak in the aqueous MCP-1 after filtration surgery corresponded to the remodeling phase, when the number of leukocytes and macrophages was decreased and fibroblasts formed the extracellular matrix. In a previous study, the number of granulocytes increased 1 day after surgery and returned to the basal level 7 days after surgery in a mouse model of conjunctival scarring [24]. In that study, the number of mononuclear cells peaked at 2 days and then decreased to the basal level 14 days after surgery, and only the number of fibroblasts remained elevated 14 days after surgery. Given that corneal and conjunctival fibroblasts produce various cytokines/chemokines [25], fibroblasts might contribute to the elevated aqueous MCP-1 level 2 weeks after filtration surgery.

The aqueous MCP-1 level was significantly higher 6 and 12 h after surgery in the trabeculectomy group than in non-operated eyes, whereas a significant difference was observed only at 3 h during the corresponding period after surgery in the Ex-PRESS group. Strikingly, the mean concentration at 12 h in the Ex-PRESS group was half of that in the trabeculectomy group (932.5 ± 531.2 vs. 1899.8 ± 515.0 pg/mL), although the difference was not significant (P = 0.109). This difference might partly explain the rapid recovery of vision after Ex-PRESS tube shunt surgery compared to trabeculectomy. Taken the difference in bleb-wall intensity between the groups into consideration, there might be small differences in the molecular mechanisms of the wound-healing process between the groups. Thus, though the post-surgical IOP was controlled similarly in this basic study and past clinical studies [9,16–19], the optimal manner of post-surgical treatment to keep adequate IOP level might be different between the groups.

What subclinical mechanisms, if any, do offset the advantages of Ex-PRESS tube shunt surgery described above, including the time-dependent changes in the aqueous MCP-1 concentration, leading to the similar surgical success rates to trabeculectomy? One possibility is foreign-body reaction to the Ex-PRESS tube made of implantable stainless steel. However, the implantation of this device at the corneoscleral junction of the rabbit eye resulted in minimal inflammatory and scarring reactions [26], suggesting the tissue response to Ex-PRESS tube is not extensive. Alternative hypothesis would be that the relatively small filtering volume of aqueous humor at early stage after surgery, which was suggested by the lower ratio of postoperative hypotony [7,16–18], might drive down the surgical success rate at later stage. Interestingly, it was reported that the mean IOP on postoperative days 9 ± 14 was significantly correlated with the surgical outcome after trabeculectomy, and a receiver operating characteristic plot suggested that mean IOP of 8 mm Hg in this period would give an optimum balance [27]. Thus, controlled filtering volume of aqueous humor after Ex-PRESS tube shunt surgery might provide multifaceted effects on wound healing mechanisms and surgical results.

This study was limited by the relatively small sample size and the 3-month observation period. The fact that we studied rabbits must also be considered when discussing the clinical relevance of our results. In addition, the rabbits used in this study were not glaucoma model, and therefore the pathophysiological backgrounds of the rabbits were different compared to human surgical cases.

In conclusion, the postoperative appearance of blebs, IOP control, and immunohistochemical findings were similar between the Ex-PRESS and trabeculectomy groups. The density of the bleb wall in the anterior-segment OCT images was significantly lower 1 week after surgery in the Ex-PRESS group compared to the trabeculectomy group. The postoperative aqueous MCP-1 level showed bimodal peaks in both groups, and there were no significant differences at any time point between the groups.
